# Neural tube defects and uterus development in human fetuses

**DOI:** 10.1038/s41598-022-18431-1

**Published:** 2022-08-18

**Authors:** André L. L. Diniz, Rodrigo R. Vieiralves, Francisco J. B. Sampaio, Carla M. Gallo, Luciano Alves Favorito

**Affiliations:** Urogenital Research Unit, State University of Rio de Janeiro, Rua Professor Gabizo, 104/201, Tijuca, Rio de Janeiro, RJ CEP 20271-320 Brazil

**Keywords:** Anatomy, Urology

## Abstract

One of the most common malformations of the central nervous system is related to embryonic neural tube alterations. We hypothesized that anencephaly affects the development of the uterus during the human second trimester of pregnancy. The objective of this study was to study the biometric parameters of the uterus in fetuses with anencephaly and compare them with normocephalic fetuses at that important. In our study, 34 female fetuses were analyzed, 22 normal and 12 anencephalic, aged between 12 and 22 weeks post-conception (WPC). After dissection of the pelvis and individualization of the genital tract, we evaluated the length and width of the uterus using the Image J software. We compared the means statistically using the Wilcoxon-Mann–Whitney test and performed linear regression. We identify significant differences between the uterus length (mm)/weight (g) × 100 (p = 0.0046) and uterus width (mm)/weight (g) × 100 (p = 0.0013) when we compared the control with the anencephalic group. The linear regression analysis indicated that 80% significance was found in the correlations in normocephalic fetuses (12.9 to 22.6 WPC) and 40% significance in anencephalic fetuses (12.3 to 18.6 WPC). The measurements of the uterus were greater in anencephalic group but there are no difference in the uterine width and length growth curves during the period studied. Further studies are required to support the hypothesis suggesting that anencephaly may affect uterine development during the human fetal period.

## Introduction

Neural tube defects (NTDs) are one of the most common congenital malformations of the central nervous system, with an average prevalence at birth of 1 in 1000^1^. Anencephaly is the most severe NTD, resulting from failure of the neural tube in the third to fourth week (days 26 to 28) after conception^2^. The possibility of secondary effects of anencephaly on other organs and systems, have motivated studies to better understand that condition.

Recently, the urogenital tract of anencephalic fetuses has been studied, including the kidneys and collecting system^2,3^, ureters^4^, bladder^5^, prostate^6,7^, penis^8^, testicles^9^, female external genitalia^10^ and vagina^11^. Some authors have indicated a different response of male and female anencephalic fetuses to hormonal stimulation^12^. One of the reasons for this difference was explained in articles describing the underdevelopment of the adrenal cortex in anencephalic fetuses^13–15^, since that segment of adrenal tissue is also responsible for the production of virilizing hormones. In the absence of this signaling, an XY fetus would not achieve full growth of its sexual characteristics. Thus, in theory anencephaly would not negatively influence the development of gynecological structures, including the uterus from a neuroendocrine perspective. But from another point of view, NTD can affect the development of the nerve plexuses that surround the uterus.

The uterus is derived from the paramesonephric (or Müllerian) ducts. By the eighth week, the paramesonephric ducts merge in the cranio-caudal direction and at the end of the first-trimester, development of the uterus and the other Müllerian structures is complete^16,17^**.** Research of the effect of anencephaly on uterine morphogenesis is still scarce.

We hypothesized that anencephaly impacts the uterine development during the human fetal period. The confirmation of these alterations could be important in future studies about the impact of NTDs on female genital development. The objective of this study was to compare the uterus diameters in fetuses with anencephaly and compare them with the biometric parameters of normocephalic fetuses at different gestational ages.

## Material and methods

The fetuses used in this study (both Controls and with AWDs all with informed consent of the parents) were obtained from the Department of Pathology of the Fernandes Figueira Institute, Oswaldo Cruz Foundation, Ministry of Health, in partnership with our University, via an official Cooperation Term.

The study was approved by the Ethical Committee on Human Research—University Hospital of the State University of Rio de Janeiro (CEP / HUPE), with the number (IRB: 2.475.334, CAAE: 78881317.4.0000.5259).

The study has also been registered in the Brazil Plataform, Ministry of Health, National Health Council, National Research Ethics Commission (CONEP) for studies with human beings. We confirm that all methods used in this paper were carried out in accordance with relevant guidelines and regulation.

Thirty-four female fetuses (22 without apparent anomalies and 12 anencephalic) were studied, aged 12 to 22 weeks post-conception (WPC), which had been aborted because of hypoxia. All of them were macroscopically well preserved and were donated by the hospital’s obstetrics department.

The gestational age was determined in WPC according to the foot-length criterion. This criterion is currently considered the most acceptable parameter to estimate gestational age^18–20^. The fetuses were also evaluated regarding, crown-rump length (CRL) and body weight immediately before dissection. All measurements were carried out by the same observer.

After compiling anthropometric data, specimens were thoroughly dissected through bilateral subcostal incision laparotomy, allowing visualization of abdominal organs and extraction of fetal pelvis “en bloc”.

The pelvis blocks were then reserved in an formaldehyde prefilled container until the moment of microdissection, performed in our laboratory with aid of stereoscopic magnification lenses (Zeiss Discovery V8 microscope 16/25×). All fetuses were dissected under identical conditions by the same researcher, who has practical experience in microsurgery.

**Figure 1:**
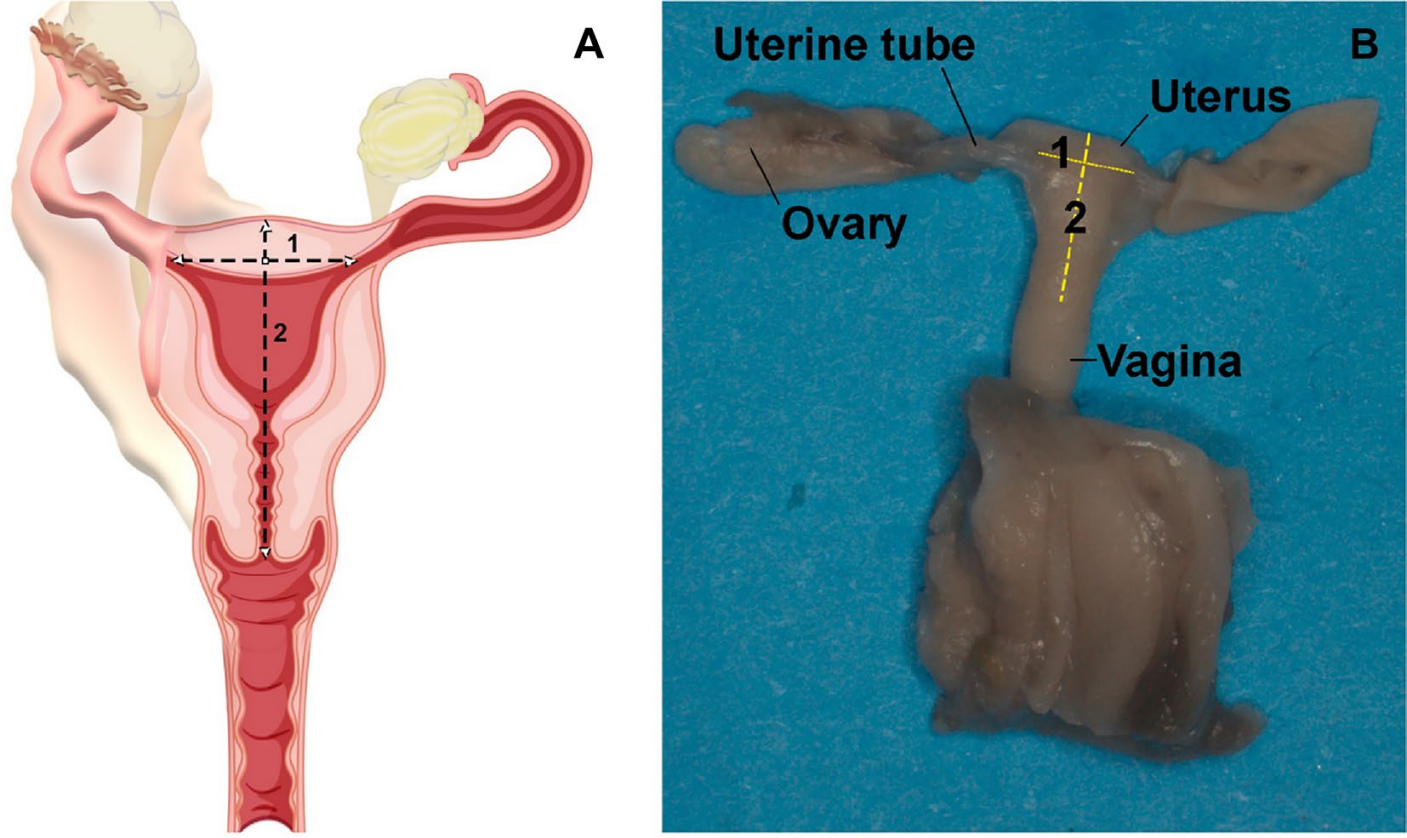
Measurements of uterus in human fetuses. (**A**) Schematic drawing of female organs showing the metric standards used to measure the uterus width (1) and uterus length (2) and (**B**) genital organs of a female fetus with 18 weeks post conception, after the dissection with the aid of the microscope (with ×16/25 magnification). The measurement of uterus width (1) and uterus length (2) was done using the Image J software, version 1.46r.

The pelvis was opened to expose and identify the urogenital organs and separate the genital and urinary tracts. After complete dissection of the uterus, photographs were taken by the camera attached to the microscope (Zeiss Axiocam 506 Color, 6 megapixels), and images were stored in a TIFF file. The biometric parameters were recorded, with measurements performed by the same observer using the Image J software, version 1.46r, because of the high intra observer precision compared to interobserver analysis^21^. Uterine dimensions were measured assuming the length from the cervix to the fundus and its width was equal to the distance between the utero-tubal junctions (Fig. 1). The data were expressed in millimeters. All data were collected from July 2019 to December 2021.

### Statistical analysis

All parameters were statistically processed and graphically described. The Shapiro–Wilk test was used to verify the normality of the data. After that, the Wilcoxon-Mann–Whitney test was used for comparison of quantitative data of normocephalic fetuses vs. fetuses with anencephaly (*p* < 0.05) and the level of significance was adjusted by the correction of Bonferroni.

Simple linear correlations (r^2^ values less than 0.4 reflect very weak correlation, while r^2^ between 0.4 and 0.7 reflect moderate correlation and r^2^ greater than 0.7 indicates strong correlation) were calculated for uterine measurements, according to fetal age. The statistical analysis was performed with the GraphPad Prism software (Version 9.2.0).

### Compliance with ethical standards

This study was supported by the National Council for Scientific and Technological Development (CNPQ—Brazil) (Grant number: 301522/2017) and The Rio de Janeiro State Research Foundation (FAPERJ) (Grant number: E26/202.873/2017).

### Ethical approval

This study was carried out in accordance with the ethical standards of the hospital’s institutional committee on human experimentation. (IRB: 2.475.334, CAAE: 78881317.4.0000.5259).

### Statement

We confirm that all data generated or analysed during this study are included in this published article submitted to *Scientific Reports*.

## Results

All biometric data of the 34 fetuses of the control and anencephalic group are reported in Table 1. The statistical analysis of all fetal biometric parameters and uterine measurements is reported in Table 2.

**Table 1:** The table shows the different parameters analyzed in the 34 fetuses studied.

Fetuses	Age (WPC)	Weight (g)	CRL (cm)	Uterus length (cm)	Uterus width (cm)
Normal	12.90	58.00	9.00	2.02	1.99
Normal	13.60	100.00	12.00	2.87	2.58
Normal	13.80	76.00	11.50	2.10	2.65
Normal	14.50	100.00	12.50	2.21	2.49
Normal	14.90	196.00	14.00	3.32	4.16
Normal	15.10	122.00	12.00	2.13	3.51
Normal	15.20	124.00	15.00	2.10	2.96
Normal	16.60	198.00	17.00	2.92	3.42
Normal	16.70	134.00	11.00	2.61	2.55
Normal	16.80	140.00	12.50	3.56	5.33
Normal	16.80	78.00	9.50	3.21	3.18
Normal	17.50	142.00	12.50	5.45	3.74
Normal	17.50	344.00	19.00	6.36	7.91
Normal	17.70	144.00	13.00	6.03	5.66
Normal	17.90	30.00	7.50	2.86	4.64
Normal	18.60	306.00	17.00	2.42	4.14
Normal	18.70	78.00	11.00	2.92	3.46
Normal	19.20	196.00	16.50	3.20	4.07
Normal	20.10	262.00	18.00	5.99	3.68
Normal	21.50	326.00	20.00	5.49	4.07
Normal	21.70	252.00	17.00	5.32	4.63
Normal	22.60	348.00	19.50	3.83	3.75
Anencephalic	12.30	30.00	8.00	2.09	3.16
Anencephalic	12.30	30.00	7.50	1.91	2.54
Anencephalic	13.60	46.00	10.00	1.88	4.68
Anencephalic	14.00	22.00	7.00	3.58	3.67
Anencephalic	14.30	76.00	10.50	2.21	2.52
Anencephalic	14.50	30.00	8.00	1.71	2.08
Anencephalic	16.30	34.00	8.00	1.81	4.22
Anencephalic	16.50	136.00	14.00	2.57	3.60
Anencephalic	17.60	28.00	7.50	5.44	2.79
Anencephalic	18.00	188.00	12.50	4.46	6.48
Anencephalic	18.30	24.00	8.00	4.17	2.88
Anencephalic	18.60	315.00	16.00	3.86	5.13

*CRL* crown-rump length, *WPC* weeks post-conception.

**Table 2:** The table shows the statistical analysis of the biometric parameters studied in 34 female fetuses, 22 normal and 12 anencephalic, aged between 12 and 22 weeks post-conception (WPC).

	Groups										p-value
	Normal					Anencephalic					
	Minimum	Maximum	Mean	Median	Standard deviation	Minimum	Maximum	Mean	Median	Standard deviation	
Age (WPC)	12.900	22.600	17.268	17.150	2.675	12.300	18.600	15.525	15.400	2.307	0.0819
Weight (g)	30.000	348.000	170.636	141.000	97.074	22.000	315.000	79.917	32.000	90.462	0.0017*
Crown-rump length (cm)	7.500	20.000	13.955	12.750	3.579	7.000	16.000	9.750	8.000	2.943	0.0015*
Uterus length (mm)	2.020	6.360	3.587	3.060	1.465	1.710	5.440	2.974	2.390	1.268	0.1487
Uterus width (mm)	1.990	7.910	3.844	3.710	1.296	2.080	6.480	3.646	3.380	1.284	0.6004
Uterus length (mm)/weight (g) × 100	0.791	9.533	2.695	2.161	1.810	1.225	19.429	7.493	5.512	6.440	0.0046*
Uterus width (mm)/weight (g) × 100	1.078	15.467	3.121	2.439	2.924	1.629	16.682	8.184	9.215	4.658	0.0013*

*SD* standard deviation.

*Significant statistical difference.

The gestational age of fetuses ranged from 12 to 22 weeks post-conception (WPC). The normocephalic group’s average gestational age was 17 WPC while for the anencephalic group it was 15 WPC. This difference was not statistically significant (*p*-value 0.0819).

The fetuses’ weight ranged from 22 to 248 g (g). The normocephalic group’s average weight was 170.64 g while for the anencephalic group it was 79.917 g. This difference was statistically significant (*p*-value 0.0017).

The measurement of the crown-rump length (CRL) of the fetuses in the whole sample ranged from 7 to 20 cm (cm). The normocephalic group’s average CRL was 13.95 cm, while for the anencephalic group it was 9.75 cm, a difference that was statistically significant (*p-*value 0.0015).

We did not identify statistical significance between the groups for the measurements of uterus length (Control: 2.02–6.36 mm/mean = 3.59 mm/SD+ −1.43 vs. Anencephalic: 1.71–5.44 mm/mean = 2.97 mm/SD + −1.21, p = 0.1070) and uterus width (Control: 1.99–7.91 mm/mean = 3.84 mm/SD+ −1.27 vs. Anencephalic: 2.08–6.48 mm/mean = 3.65 mm/SD+ −1.23; *p* = 0.3360).

When the uterine length and weight data were normalized to the fetal weight in each case, and then compared we calculate two new variables: uterine length/fetal weight × 100 and uterine width/fetal weight × 100. We identify significant differences between the uterus length (mm)/weight (g) × 100 (p = 0.0046) and uterus width (mm)/weight (g) × 100 (p = 0.0013) when we compare the control with the anencephalic group. The summary of the findings regarding the correlations studied between the uterus length (mm)/weight (g) × 100 (p = 0.0046) and uterus width (mm)/weight (g) × 100 (p = 0.0013) in the normal and anencephalic groups is reported in Table 2.

The summary of the findings regarding the correlations studied in the normal and anencephalic groups is reported in Table 3. The linear regression analysis indicated that 80% significance was found in the correlations in normocephalic fetuses (12.9 to 22.6 WPC) and 40% significance in anencephalic fetuses (12.3 to 18.6 WPC) during the period studied.

**Table 3:** The table shows the coefficient and significance of the correlations studied in normal and anencephalic fetuses.

	Normal			Anencephalic		
	r^2^	*p*-value		r^2^	*p*-value	
Age (WPC) × weight	r^2^ = 0.4677	*p* = 0.004	Significant	r^2^ = 0.3101	*p* = 0.0600	Not Significant
Age (WPC) × CRL	r^2^ = 0.3986	*p* = 0.0016	Significant	r^2^ = 0.2426	*p* = 0.1038	Not Significant
Age (WPC) × uterus length	r^2^ = 0.3507	*p* = 0.0037	Significant	r^2^ = 0.5218	*p* = 0.0080	Significant
Age (WPC) × uterus width	r^2^ = 0.1590	*p* = 0.0660	Not Significant	r^2^ = 0.1891	*p* = 0.1577	Not Significant
Weight × CRL	r^2^ = 0.8862	*p* < 0.0001	Significant	r^2^ = 0.8723	*p* < 0.0001	Significant
Weight × uterus length	r^2^ = 0.3276	*p* = 0.0054	Significant	r^2^ = 0.08606	*p* = 0.3547	Not Significant
Weight × uterus width	r^2^ = 0.2499	*p* = 0.0178	Significant	r^2^ = 0.4313	*p* = 0.0203	Significant
CRL × uterus length	r^2^ = 0.2735	*p* = 0.0125	Significant	r^2^ = 0.02097	*p* = 0.6534	Not Significant
CRL × uterus width	r^2^ = 0.1572	*p* = 0.0677	Not Significant	r^2^ = 0.3493	*p* = 0.0430	Significant
Uterus width × uterus length	r^2^ = 0.4607	*p* = 0.0005	Significant	r^2^ = 0.08365	*p* = 0.3619	Not Significant

**Figure 2:**
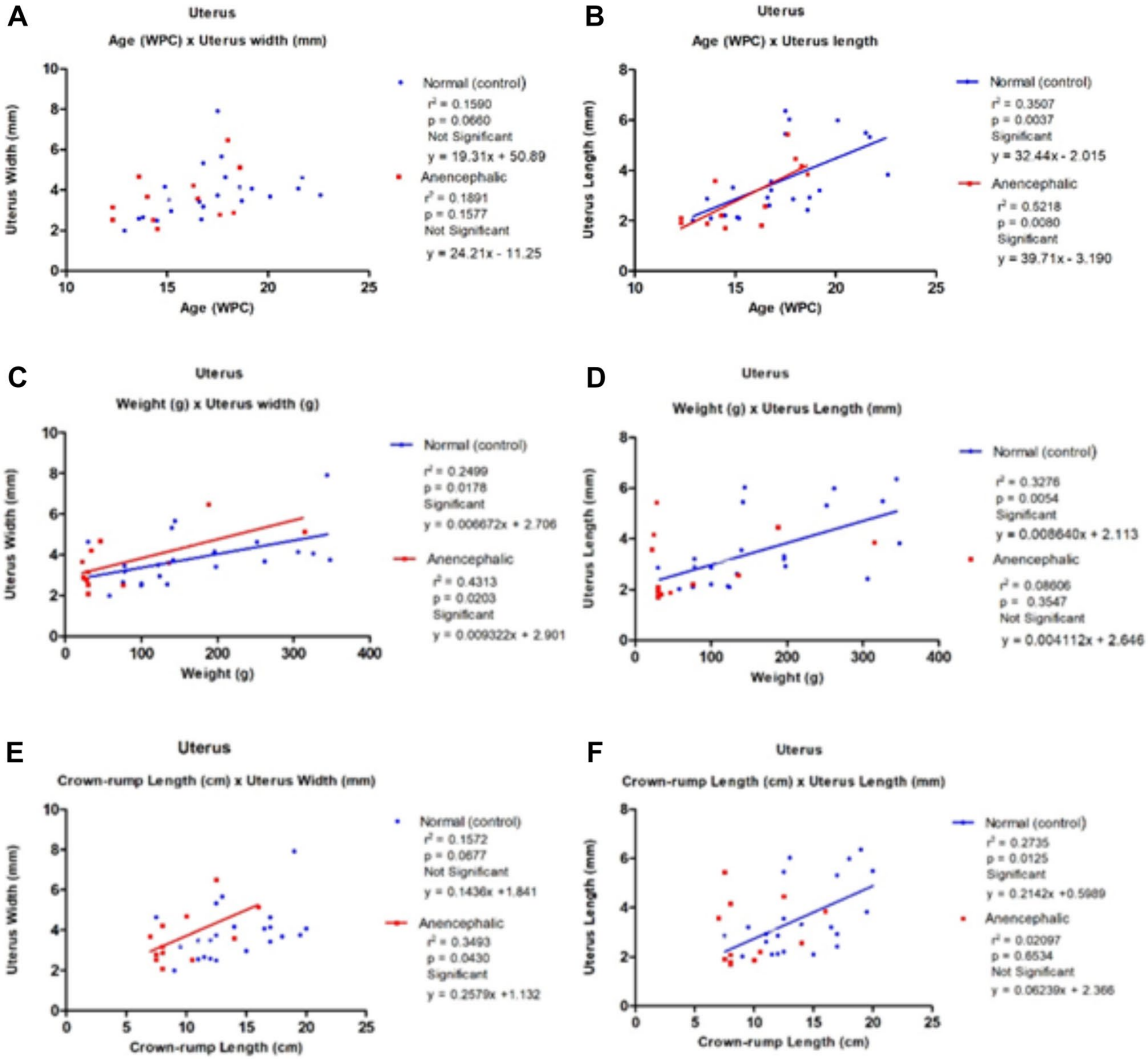
The figure shows the linear regression analysis comparing the biometric data of the uterus with fetal age (WPC), weight (g) and crown-rump length (cm). (**A**) Uterus width × fetal age: the linear regression analysis shows non-significant correlation between fetal age and uterine width in normal and anencephalic groups; (**B**) uterus length × fetal age: the linear regression analysis shows a significant and positive correlation between fetal age and uterine length in normal and anencephalic groups; (**C**) uterine width × fetal age: the linear regression analysis shows a significant and positive correlation between fetal weight and uterine width in the normal and anencephalic groups; (**D**) uterine length × fetal weight: the linear regression analysis shows a significant and positive correlation between fetal weight and uterine length in the normal group, but not significant in the anencephalic group; (**E**) uterine width × crown-rump length: the linear regression analysis shows a significant and positive correlation between fetal weight and uterine length in the anencephalic group, but not significant in the normal group and (**F**) uterine length × crown-rump length: the linear regression analysis shows a significant and positive correlation between fetal weight and uterine length in the normal group, but not significant in the anencephalic group.

The gestational age (WPC) was correlated with the length of the uterus (mm) in the control group (12.9–22.6 WPC) (*y* = 32.44*x* − 2.015) and anencephalic group (12.3–18.6 WPC) (*y* = 39.71*x* – 3.190). The results showed that gestational age was significantly and positively correlated with the uterine length of the normal (control) and anencephalic fetuses. The gestational age (WPC) was correlated with the width of the uterus (mm) in the control group (12.9–22.6 WPC) (*y* = 19.31*x* + 50.89) and anencephalic group (12.3–18.6 WPC) (*y* = 24.211*x* – 11.25). The results showed that the correlation of gestational age with the uterine width of the normal (control) and anencephalic fetuses was not statistically significant (Fig. 2 and Table 3).

The fetal weight was correlated with the length of the uterus (mm) in the control group (12.9–22.6 WPC) (*y* = 0.00864*x* + 2.113) and anencephalic group (12.3–18.6 WPC) (*y* = 0.004112*x* + 2.646). The results showed that fetal weight was significantly and positively correlated with the uterine length of the normal group, but not for the anencephalic group. The fetal weight was correlated with the width of the uterus (mm) in the control group (12.9–22.6 WPC) (*y* = 0.006672*x* + 2.706) and anencephalic group (12.3–18.6 WPC) (*y* = 0.009322*x* + 2.901). The results showed that fetal weight was significantly and positively correlated with the uterine length of the normal group and the anencephalic group (Fig. 2 and Table 3).

The CRL (cm) was correlated with the length of the uterus (mm) in the normal fetuses (12.9–22.6 WPC) (*y* = 0.2142*x* + 0.5989) and anencephalic fetuses (12.3–18.6 WPC) (*y* = 0.06239*x* + 2.366). The results showed that CRL was significantly and positively correlated with the uterine length of the normal group, but not of the anencephalic group. The CRL was correlated with the width of the uterus (mm) in normal fetuses (12.9–22.6 WPC) (*y* = 0.1436*x* + 1.841) and anencephalic fetuses (12.3–18.6 WPC) (*y* = 0.2579*x* + 1.132). CRL was significantly and positively correlated with the uterine width of the anencephalic group, but not of the normal group (Fig. 2 and Table 3).

## Discussion

In normal fetuses, GnRH-containing neurons are present in the human brain at the end of the first month of pregnancy^22^. Hypophysis is able to synthesize gonadotropins from at least 9 weeks of fetal life^23,24^, so the release of gonadotropins into the fetal circulation can be demonstrated by the beginning of the second trimester. Circulating luteinizing hormone (LH) and follicle stimulating hormone (FSH) levels were significantly higher in female than in male fetuses at mid-gestation^25^.

Differentiation of the genital tract depends on metabolic pathways initially orchestrated by the presence/absence of the SRY gene and by the action of anti-Müllerian hormones (AMH) and testosterone. Thus, it is expected that a female embryo, genetically XX, will not susceptible to the direct action of anti-Müllerian hormones (AMH) and testosterone.

Fetal ovaries are unable to secrete AMH, but their ovarian expression can be detected by immunohistochemistry at the time of the third trimester, suggesting that AMH plays a role in ovarian development as early as the fetal period^16,26^. Circulating FSH concentrations in female fetuses are high at mid-gestation, then decrease to low levels at birth, but transiently increase again during postnatal pituitary activation^27^. In premature girls, extremely high postnatal levels of FSH have been described, indicating an alteration in pituitary-ovarian function in infancy^27,28^.

In this sense, since the exposure of the central nervous system to amniotic fluid causes irreversible damage to the encephalic tissue, we could assume that the primary hormonal axis would be compromised in anencephalic fetuses, which could reflect the abnormal development of the reproductive organs^1,9^.

This correlation would be more assertive for male fetuses since the fetal ovaries are not thought to play any part in female sex differentiation, because they secrete little estrogen, even though follicles begin to develop at about 16 weeks and primordial follicles containing granulosa cells are present by 20 weeks^16^. It has been described that female external genitalia development is not subject to fetal gonadal hormones as in male fetuses^29,30^.

In male fetuses, the comparative study of the development of the genital tract of normal and anencephalic fetuses has been conducted over the years. Zondek & Zondek, during the 1960s, observed that the prostate showed marked, and in some instances extreme, metaplastic changes, sometimes even surpassing the appearance in normal controls^7^. In another paper, those researchers noted that the volume of the testis was smaller than that of controls with similar periods of development^12^.

In our department, Pires et al. observed that the testicular volume of anencephalic fetuses did not increase with fetal age, and developed more slowly than in normal fetuses. On the other hand, the same research team did not observe significant differences in development of the prostate in fetuses with anencephaly^6,9^. In the same sense, Carvalho and colleagues, using histological and immunolabeling techniques, concluded that anencephaly does not cause structural alterations in the fetal penis^8^.

As for the female fetuses, Baker and Scrimgeour demonstrated that gonadal development was almost identical in the ovaries of anencephalic and control fetuses^31^. Zondek and Zondek reported a different result, suggesting that the volume of the ovary in anencephalics was larger than that of the controls up to 36 weeks of gestation and somewhat smaller in the last month of pregnancy; with no marked degree of hypoplasia^12^.

Previous data from our department showed no differences in vaginal morphology, but the vaginal length and width were smaller in the anencephalic group during the second trimester of pregnancy^11^. Changes were perceived in relation to the external genitalia, with anencephalic fetuses tending to have more rudimentary external genitalia, with a reduction in anatomical distances from some reference points: length and width of the clitoris, length and width of the vaginal introitus, and distance between the labia majora and the clitoris-anus distance. Despite these findings, there was no significant change in the distance between the vaginal opening and the anus of these fetuses^10^.

Comparative studies of the uterus between normal and anencephalic fetuses are scarce in the literature. Zondek and Zondek evaluated genital structures of female anencephalic fetuses, stillborn in the third trimester. Their histological investigation demonstrated normal uterine development, with vascular, stromal and glandular proliferation, glandular secretion present in varying degrees, and full development during the last month of pregnancy^32^.

The present paper reports the first normative parameters of uterine development during the second gestational trimester in human fetuses. The statistical analysis of our measurements indicates that the uterine diameters were different between the evaluated groups. The measurements of the uterus were significantly greater in anencephalic group and the linear regression analysis indicated that 80% significance was found in the correlations in control group and 40% significance in anencephalic fetuses but without statistical difference in the uterine width and length growth curves during the 2nd gestational trimester. We can speculate that NTDs could have impact on uterine development during the human gestational period probably because of commitment of the primary hormonal axis in anencephalic fetuses and the consequent alteration in the growth of organs of the genital system in fetuses as evidenced in previous studies^5,9,10^. However we need more studies including ultrastructural and sophisticated histological analysis, providing better assessment of these changes in fetuses of the 3rd gestational trimester to support this hypothesis.

Uterine development could be influenced by other factors independently of anencephaly. It is assumed that the Vangl2 gene is vital for neural tube closure (mainly a recessive effect) and independently for female reproductive tract development (a dominant effect). In a previous experimental study, heterozygotes for the Vangl2-Lp mutation develop mild spinal neural tube defects but also show a high frequency of imperforate vagina^33^. Moreover, it is important to make clear that the case of Vangl2 mutants in mice is just an example, where the primary NTD effect appears separate from the effect on the female genital tract. There may well be other genes or environmental factors that also have a similar effect.

Some limitations of our study should be mentioned: (a) the unequal WPC of the anencephalic fetuses and the control group; (b) the lack of 3rd trimester fetuses to confirm the uterine growth pattern in the two groups studied; (c) the lack of uterine histopathological analysis in our sample; (d) the small sample size (however, anencephalic fetuses are rare, so the observations of a small sample are still relevant); and (e) measurement of uterine biometric parameters by a single observer, potentially leading to measurement bias.

## Conclusions

The comparative study of uterine dimensions between normal and anencephalic fetuses indicated differences between the two groups. The measurements of the uterus were greater in anencephalic group but there are no difference in the uterine width and length growth curves during the period studied. Further studies are required to support the hypothesis suggesting that anencephaly may affect uterine development during the human fetal period. We reiterate that the translational aspects of the anencephalic model are promising in the fields of embryology, fetal medicine, neonatology and pediatric urology.

## Abbreviations

NTDs: Neural tube defects
WPC: Weeks post-conception
CRL: Crown-rump length
LH: Luteinizing hormone
FSH: Follicle stimulating hormone
AMH: Anti-Müllerian hormones
CAAE: Certificate of Ethical Appreciation Presentation
CNPQ: National Council for Scientific and Technological Development
FAPERJ: Rio de Janeiro State Research Fundation
IRB: Institutional Review Board
mm: Millimeters
mm^2^: Square millimeters
p: P-value
r^2^: Pearson correlation coefficient
SD: Standard deviation
Vs.: Versus
